# Attenuation of Combined Nickel(II) Oxide and Manganese(II, III) Oxide Nanoparticles’ Adverse Effects with a Complex of Bioprotectors

**DOI:** 10.3390/ijms160922555

**Published:** 2015-09-17

**Authors:** Ilzira A. Minigalieva, Boris A. Katsnelson, Larisa I. Privalova, Marina P. Sutunkova, Vladimir B. Gurvich, Vladimir Y. Shur, Ekaterina V. Shishkina, Irene E. Valamina, Oleg H. Makeyev, Vladimir G. Panov, Anatoly N. Varaksin, Ekaterina V. Grigoryeva, Ekaterina Y. Meshtcheryakova

**Affiliations:** 1 The Medical Research Center for Prophylaxis and Health Protection in Industrial Workers, 30 Popov Str., Ekaterinburg 620014, Russia; E-Mails: ilzira-minigalieva@yandex.ru (I.A.M.); privalovali@yahoo.com (L.I.P.); marinasutunkova@yandex.ru (M.P.S.); gurvich@ymrc.ru (V.B.G.); grigorieva@ymrc.ru (E.V.G.); 2 The Institute of Natural Sciences, The Ural Federal University, Ekaterinburg 620000, Russia; E-Mails: vladimir.shur@urfu.ru (V.Y.S.); ekaterina.shishkina@labfer.usu.ru (E.V.S.); 3 The Central Research Laboratory, The Ural State Medical University, 17 Klyuchevskaya Str., Ekaterinburg 620109, Russia; E-Mails: ivalamina@mail.ru (I.E.V.); ommt305@mail.ru (O.H.M.); katusha-ugma@rambler.ru (E.Y.M.); 4 Institute of Industrial Ecology, the Urals Branch of the Russian Academy of Sciences, 20 Sofia Kovalevskaya Str., Ekaterinburg 620990, Russia; E-Mails: vpanov@ecko.uran.ru (V.G.P.); varaksin@ecko.uran.ru (A.N.V.)

**Keywords:** nanoparticles, manganese(II, III) oxide, nickel(II) oxide, subchronic toxicity, bioprotectors

## Abstract

Stable suspensions of NiO and Mn_3_O_4_ nanoparticles (NPs) with a mean (±s.d.) diameter of 16.7 ± 8.2 and 18.4 ± 5.4 nm, respectively, purposefully prepared by laser ablation of 99.99% pure nickel or manganese in de-ionized water, were repeatedly injected intraperitoneally (IP) to rats at a dose of 2.5 mg/kg 3 times a week up to 18 injections, either alone or in combination. A group of rats was injected with this combination with the background oral administration of a “bio-protective complex” (BPC) comprising pectin, vitamins A, C, E, glutamate, glycine, *N*-acetylcysteine, selenium, iodide and omega-3 PUFA, this composition having been chosen based on mechanistic considerations and previous experience. After the termination of injections, many functional and biochemical indices and histopathological features (with morphometric assessment) of the liver, spleen, kidneys and brain were evaluated for signs of toxicity. The Ni and Mn content of these organs was measured with the help of the atomic emission and electron paramagnetic resonance spectroscopies. We obtained blood leukocytes for performing the RAPD (Random Amplified Polymorphic DNA) test. Although both metallic NPs proved adversely bio-active in many respects considered in this study, Mn_3_O_4_-NPs were somewhat more noxious than NiO-NPs as concerns most of the non-specific toxicity manifestations and they induced more marked damage to neurons in the striatum and the hippocampus, which may be considered an experimental correlate of the manganese-induced Parkinsonism. The comparative solubility of the Mn_3_O_4_-NPs and NiO-NPs in a biological medium is discussed as one of the factors underlying the difference in their toxicokinetics and toxicities. The BPC has attenuated both the organ-systemic toxicity and the genotoxicity of Mn_3_O_4_-NPs in combination with NiO-NPs.

## 1. Introduction

Nanoparticles (NPs) of metal oxides are of special interest for industrial toxicology and for occupational health risk assessment and management not only because there exists a number of purposefully manufactured (engineered) NPs of this class but also because they constitute a substantial proportion within the particle size distribution of the condensation aerosols generated by arc-welding and various metallurgical technologies. Indeed, such aerosols commonly include, along with chemically similar fine micrometer particles and submicron ones having dimensions >100 nm, the conventional nanoscale fraction (<100 nm).

The prevailing majority of the published studies have assessed the cytotoxicity and genotoxicity of NPs on stable cell lines and only rarely on laboratory animals. No doubt, *in vitro* experiments feature a number of advantages, in particular, relating to analysis of primary mechanisms of toxicity. However, any extrapolation of such experimental results to the whole mammalian organism is associated with a number of uncertainties and assumptions. Moreover, some important aspects of toxicology (in particular, toxicokinetics, dose-response relationships on organ and systemic levels, and the functioning of protective mechanisms) can generally be addressed only through experiments on a whole mammalian organism.

We have demonstrated just in such experiments that several metallic NPs are much more toxic on both *in vivo* cellular and systemic levels as compared with their one micrometer or even submicron counterparts, while the dependence of systemic toxicity on particle size within the nanometer range is non-unique due to often contra-directional relationships between the intrinsic cytotoxicity of specific nanoparticles, on the one hand, and mechanisms that control their biokinetics, on the other [1,2,3,4].

As a rule, however, the industrial aerosols generated by the above technologies (arc-welding and alloyed steel metallurgy especially) not only are polydisperse but also have a complex chemical composition comprising microparticles and nanoparticles of oxides of iron, manganese, nickel, chrome, vanadium, silicon and other elements. Both the composition of these NPs and quantitative relationships between them vary broadly depending on specific technology, its phase, composition of the molted metal, melting temperature, *etc.* One of the distinctive features of industrial nanotoxicology is, therefore, the frequent need to carry out not only separate but also comparative assessments of the toxicities of various metal oxide NPs, as well as of their combined effects. Particularly important are the manifestations of organ and systemic toxicity that are associated not only with the general mechanisms of the cytotoxic effect produced by NPs, which are very actively studied by various researchers at subcellular and cellular levels [5] focusing on the generation of reactive oxygen species and on intracellular release of metal ions, but also with the ones that are more or less specific to the toxicokinetics and toxicodynamics of certain metals.

It should not go unmentioned that the literature reports (to the best of our knowledge) very few comparative qualitative assessments of the effects exerted on the organism by chemically different NPs in parallel chronic or, at least, subchronic experiments *in vivo* for similar particle doses, shapes and sizes. We carried out a study that met these conditions for NPs of gold and silver [6]. However, it was planned not because these NPs occur in any industry and thus impact on the human organism in combination but, rather, because it was not yet quite clear theoretically which characteristics of nanomaterials play the most important role: those associated with the nano-dimension of particles of any chemical composition or those determined by the chemical nature of the NP-forming substance. The results of that study suggest that both characteristics, dimension and chemistry, are essential. If this is the case, the higher is the practical importance of the toxic effects produced by metal oxide NPs that are actually present in one and the same industrial environment.

In the context of this issue, we have chosen NPs of nickel and manganese oxides, which are often present together both in the workplace and ambient air of steel-making facilities and in welding fumes [7]. Nickel is usually present in the form of nickel(II) oxide (NiO-NPs), and manganese as a great number of oxygen compounds, one of which is manganese(II, III) oxide (Mn_3_O_4_-NPs) [8,9,10]. Experimental assessments of the toxicity of various nickel- and manganese-containing NPs, carried out on cell cultures or under intratracheal instillations, have been reported by various researchers [11,12,13,14,15,16,17,18], but, as far as we know, no comparative assessment has ever been carried out in a parallel subchronic experiment *in vivo*.

The toxic properties of various manganese and nickel oxides and salts in the form of solutions and microparticles acting alone have been extensively investigated [19,20,21]. Subchronic intoxication of rats with NiCl_2_ and KMnO_4_ acting together was characterized by different types of combined toxicity (either of additive type or departing from it predominantly towards subadditivity) depending on the effect assessed, dose, and effect level [22].

This paper, however, is focused mainly on another important but rarely considered aspect of metal nanotoxicology. We maintain that the especially high toxicity of metallic NPs calls for searching for some means to render exposed people more resistant to their adverse effects. The general concept of such “biological prophylaxis” of different occupational and environmental intoxications, its theoretical premises and general principles along with numerous examples of its practical realization have been published by us over several decades repeatedly, including in review articles [23,24]. It was but natural to explore the potential of such preventive strategy in the field of nanotoxicology as well.

Our previous experiments proved that both systemic toxicity and *in vivo* genotoxicity of silver [6] and of copper oxide [4] NPs were markedly attenuated with background oral administration of multi-component bioprotective complexes (BPC). These BPCs comprised pectin, multivitamin-multimineral preparations, some amino acids, and omega-3 PUFA. In both cases the protective action was demonstrated in relation to various adverse effects of subchronic toxicity. We also found [25] that in rats that were being given glutamate, glycine, *N*-acetyl cysteine, iodide and a Se-containing multivitamin preparation orally during 4 weeks before a single intratracheal instillation of NiO-NPs + Mn_3_O_4_-NPs (0.25 mg each), the latter evoked significantly weaker neutrophil leukocyte recruitment into the lower airways than in rats so exposed without any pretreatment. Now we are able to demonstrate the preventive efficacy of a bio-protective complex administered also orally but along with repeated i.p. injections of a similar combination of NPs.

**Table 1:** Total manganese and nickel contents of rat’s organs, mcg per g of dried tissue, after repeated intraperitoneal injections of NiO or Mn_3_O_4_ nanoparticles, together or separately, and of their combination with background oral administration of the BPC (X ± s.e.).

Metal	Groups of Rats Given (6 Rats)				
	Control	NiO-NPs	Mn_3_O_4_-NPs	NiO-NPs + Mn_3_O_4_-NPs	NiO-NPs + Mn_3_O_4_-NPs and BPC
**Liver**					
Manganese	7.33 ± 0.56	6.33 ± 0.49	7.20 ± 0.73	6.80 ± 0.20	7.17 ± 0.31
Nickel	3.17 ± 0.65	16.40 ± 3.26 *^,+,○^	2.80 ± 0.20 ^+^	38.00 ± 2.80 *	18.50 ± 1.56 *^,+^
**Spleen**					
Manganese	28.80 ± 1.74	32.00 ± 4.15	25.00 ± 1.87	25.83 ± 4.23	14.00 ± 2.33 *^,+^
Nickel	25.60 ± 1.89	46.75 ± 8.44 *	32.50 ± 4.63	36.17 ± 7.21	36.25 ± 8.97
**Kidneys**					
Manganese	13.75 ± 0.95	10.00 ± 0.63 *^,○^	20.50 ± 1.45 *^,+^	10.80 ± 1.11	16.17 ± 1.62 ^+^
Nickel	18.20 ± 1.56	20.60 ± 3.23	16.00 ± 1.58	17.60 ± 1.29	18.67 ± 1.28
**Brain**					
Manganese	6.00 ± 0.45	7.17 ± 0.98	10.75 ± 1.70 *	7.60 ± 1.08	9.20 ± 0.80 *
Nickel	14.67 ± 0.96	12.83 ± 1.20	15.75 ± 2.46	15.80 ± 2.65	8.60 ± 0.60 ^+^

* statistically significant difference from the control group; ^+^ from the group given NiO-NPs + Mn_3_O_4_-NPs (without the BPC); ^○^ from the group given Mn_3_O_4_-NPs; (*p* < 0.05 by Student’s *t*-test with Bonferroni correction).

## 2. Results

As can be seen from Table 1, the total Ni content (as determined by AES) was significantly increased under exposure to both NiO-NPs and NiO-NPs + Mn_3_O_4_-NPs in the liver and spleen only. Exposure to Mn_3_O_4_-NPs injected alone (not in combination with NiO-NPs) induced a noticeable increase in the Mn content of the kidneys and brain only. Meantime, the accumulation of Ni in the liver of the rats given this combination was significantly higher as compared with those exposed to NiO-NPs alone. This increase was not present in the group with background BPC administration, and the Ni content of the liver in this group proved 2 times lower (*p* < 0.05) as compared with the group exposed to NPs of NiO-NPs + Mn_3_O_4_-NPs without the BPC. Under the influence of the BPC, the accumulation of manganese in the spleen and nickel in the brain decreased significantly while the same BPC increased nickel accumulation in the kidneys.

**Figure 1:**
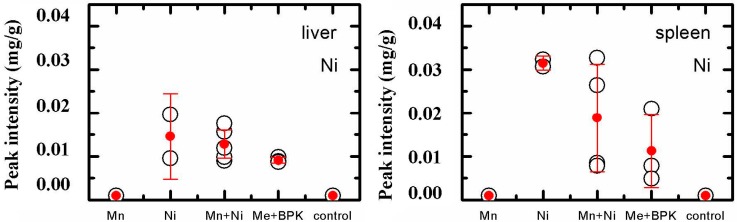
Nickel (as NiO-NPs) content of the liver and spleen in the groups of rats exposed to NiO-NPs, to Mn_3_O_4_-NPs + NiO-NPs, or to the same combination together with the BPC (designated as NiO-NPs, Mn + Ni and Me + BPC, respectively). Electron paramagnetic resonance (EPR) method. Dots correspond to arithmetic means; bars, to 95% CI.

Both NiO and Mn_3_O_4_ (and, thus, respective NPs) were detected with the help of EPR spectroscopy in the liver and spleen only, while the EPR signal of the Mn_3_O_4_-NPs was too weak for quantification. As for the NiO-NPs content (Figure 1), it was somewhat lower, rather than higher (unlike the total Ni content), in the combined exposure group compared with the group exposed to NiO-NPs alone, and again, this content was decreased by BPC administration.

As can be seen from Table 2, in the rats exposed to NiO-NPs the daily renal excretion of Ni was statistically significantly increased. The respective increase in the rats exposed to NiO-NPs + Mn_3_O_4_-NPs was also significant but half as high compared with those exposed to NiO-NPs only (*p* < 0.05), while in the rats similarly exposed to NiO-NPs + Mn_3_O_4_-NPs along with BPC administration it did not differ from the value for the NiO-NPs-exposed ones. At the same time, exposure to Mn_3_O_4_-NPs increased manganese excretion only when combined with exposure to NiO-NPs, but this increase was not significant in the rats administered the BPC.

**Table 2:** Total manganese and nickel renal excretion in rats after repeated intraperitoneal injections of NiO or Mn_3_O_4_ nanoparticles, together or separately, and of their combination with background oral administration of the BPC, mcg per 24 h (X ± s.e.).

Metal	Groups of Rats Given					
	Control (7 Rats)	NiO-NPs (8 Rats)	Mn_3_O_4_-NPs (8 Rats)	NiO-NPs + Mn_3_O_4_-NPs (7 Rats)	NiO-NPs + Mn_3_O_4_-NPs and BPC (7 Rats)	BPC (7 Rats)
Manganese	0.06 ± 0.05	0.008 ± 0.006 ^+^	0.06 ± 0.01 ^+^	0.52 ± 0.1 *	0.28 ± 0.1	0.11 ± 0.1
Nickel	2.9 ± 0.2	37.9 ± 5.3 *^,+,○^	0.63 ± 0.3 *^,+^	16.7 ± 2.9 *	40.8 ± 6.2 *^,+^	3.2 ± 0.3

* statistically significant difference from the control group; ^+^ from the group given NiO-NPs + Mn_3_O_4_-NPs (without the BPC); ^○^ from the group given Mn_3_O_4_-NPs; (*p* < 0.05 by Student’s *t*-test with Bonferroni correction).

Out of the 50 functional and biochemical indices for the organism’s status given in Table 3, a statistically significant adverse deviation from the control value was observed in 22 indices for the effect of NiO-NPs, in 26 for the effect of Mn_3_O_4_-NPs, in 25 for the combined action of the same NPs, and in only one under exposure to the same combination with the background BPC administration caused deviation in any of the indices, whereas the same administration without toxic exposure caused shifts in 6 indices (Table 3). Shifts caused by two nano-oxides under comparison were, as a rule, unidirectional and close in magnitude. However, the majority of the indices displayed a somewhat higher effect of Mn_3_O_4_-NPs than that of NiO-NPs, although in only three of them (increased liver mass, reduced number of head-dips, and reduced albumin content of the blood serum) this difference was statistically significant. In the case of combined intoxication, the majority of the indices fell within the range of values for separate exposures, though tending towards the prevalence of the low values of this range. At the same time, diuresis was lowered along with increased density of the urine only for the combined effect of the nano-oxides (differing statistically significantly from the index of not only the control group but also each of the groups exposed to one of the nano-oxides alone). The same combined exposure group displayed the highest protein and creatinine contents of the urine (with a statistically significant difference from the corresponding indices of the rats exposed to NiO-NPs alone), featuring the least endogenous creatinine clearance (significantly lower than in the group exposed to Mn_3_O_4_-NPs alone, the only group in which this index was significantly above the control value).

**Table 3:** Some functional indices for the condition of rat after repeated intraperitoneal injections of NiO or Mn_3_O_4_ nanoparticles separately and of their combination with background oral administration of the BPC (X ± s.e.).

Index	Control (10 Rats)	Mn_3_O_4_-NPs (11 Rats)	NiO-NPs (11 Rats)	NiO-NPs + Mn_3_O_4_-NPs (12 Rats)	NiO-NPs + Mn_3_O_4_-NPs and BPC (12 Rats)	BPC (10 Rats)
Starting body mass, g	195.0 ± 4.8	207.3 ± 4.9	201.0 ± 3.0	203.2 ± 3.0	200.0 ± 5.9	204.5 ± 5.4
Final body mass, g	232.5 ± 5.5	243.2 ± 4.6	244.0 ± 2.9	247.3 ± 6.6	238.6 ± 7.6	239.5 ± 5.5
Body mass gain, %	19.4 ± 2.1	17.5 ± 1.4	21.5 ± 1.3	21.6 ± 2.0	19.4 ± 2.3	17.3 ± 1.4
Liver mass, g per 100 g body mass	3.5 ± 0.1	4.8 ± 0.1 *^,+^	4.0 ± 0.1 *^,○^	3.9 ± 0.1	3.7 ± 0.2	3.7 ± 0.2
Spleen mass, g per 100 g body mass	0.36 ± 0.01	0.48 ± 0.03 *	0.41 ± 0.02	0.44 ± 0.03 *	0.37 ± 0.03	0.38 ± 0.02
Kidney mass, g per 100 g body mass	0.64 ± 0.01	0.62 ± 0.02 *	0.59 ± 0.01 *	0.59 ± 0.01 *	0.58 ± 0.01	0.64 ± 0.02
Brain mass, g per 100 g body mass	0.76 ± 0.01	0.72 ± 0.01 *	0.72 ± 0.02	0.7 ± 0.02	0.68 ± 0.02	0.77 ± 0.02
Temporal summation of sub-threshold impulses, s.	15.9 ± 0.6	17.2 ± 1.03	17.6 ± 1.03	16.7 ± 1.0	16.9 ± 0.9	16.9 ± 1.1
Number of head-dips into holes during 3 min	9.2 ± 0.9	2.3 ± 0.6 *^,+^	5.3 ± 1.1 *^,○^	6.0 ± 1.0 *	3.3 ± 1.4 *	5.4 ± 1.5
Hemoglobin, g/dL	14.0 ± 0.4	13.0 ± 0.4	12.6 ± 0.2 *	12.0 ± 0.8 *	13.0 ± 0.4	13.8 ± 0.2
Erythrocytes, 10^12^ cells/L	7.8 ± 0.2	5.8 ± 0.2 *	5.8 ± 0.2 *	5.6 ± 0.4 *	6.2 ± 0.2	6.4 ± 0.2
Average volume of red blood cells, µm^3^	61.5 ± 1.0	65.9 ± 1.0 *	64.6 ± 1.0 *	64.9 ± 1.0 *	61.8 ± 0.9 ^+^	65.9 ± 0.8 *
Hematocrit, %	42.6 ± 0.6	38.4 ± 1.2 *	37.8 ± 1.0 *	36.6 ± 1.0 *	39.4 ± 1.0	41.4 ± 1.0
Reticulocytes, ‰	21.4 ± 3.1	25.2 ± 3.7	31.1 ± 3.1 *	27.3 ± 3.0	21.5 ± 2.3	17.5 ± 2.3
Leukocytes, 10^3^/µL	8.6 ± 0.8	10.4 ± 0.6	8.4 ± 0.8 ^+^	12.2 ± 1.0*	11.4 ± 1.2 ^+^	8.6 ± 0.8
Thrombocytes, 10^3^/µL	735.2 ± 37.6	771.6 ± 41.0	718.2 ± 36.8	802.6 ± 30.2	776.4 ± 39.8	751.4 ± 36.0
Lymphocytes,%	93.7 ± 1.0	84.5 ± 2.0 *^,+^	85.6 ± 2.3 *	90.5 ± 1.5	91.3 ± 1.2	93.2 ± 0.8
Monocytes,%	4.9 ± 0.9	12.2 ± 1.6 *^,+^	11.1 ± 2.0 *	7.7 ± 1.3	7.2 ± 0.9	5.4 ± 0.7
Granulocytes,%	1.3 ± 0.2	3.3 ± 0.6 *^,+^	3.2 ± 0.4 *^,+^	1.8 ± 0.3	1.6 ± 0.2	1.4 ± 0.3
Bilirubin in blood serum, μmol/L	2.02 ± 0.4	0.97 ± 0.1 *	1.15 ± 0.1 *	1.15 ± 0.1 *	1.5 ± 0.1 ^+^	1.7 ± 0.1
Succinate dehydrogenase (SDH) activity, number of formazane granules per 50 lymphocytes	647.1 ± 14.5	551.6 ± 20.6 *	560.7 ± 11.5 *	530.2 ± 22.5 *	569.1 ± 17.6	591.0 ± 11.5 *
Total protein content of blood serum, g/L	76.0 ± 1.3	75.2 ± 1.7^+^	71.2 ± 1.7 *	69.4 ± 1.9 *	72.7 ± 1.2	79.4 ± 1.5
Albumin content of blood serum, g/L	46.6 ± 0.8	38.3 ± 0.9 *	42.5 ± 1.1 *^,+,○^	38.6 ± 0.8 *	41.8 ± 1.1 ^+^	47.3 ± 1.2
Globulins of blood serum, g/L	29.4 ± 0.9	36.9 ± 1.5 *^,+^	28.8 ± 1.6 ^○^	30.7 ± 1.4	30.9 ± 0.9	32.1 ± 1.2
A/G index	1.6 ± 0.06	1.1 ± 0.05 *^,+^	1.5 ± 0.1 ^+,○^	1.3 ± 0.04 *	1.4 ± 0.1	1.5 ± 0.1
AST activity in blood serum, mM/h × L	378.5 ± 25.7	275.0 ± 25.2 *	279.4 ± 24.6 *	279.0 ± 18.4 *	338.8 ± 43.5	305.8 ± 18.5 *
ALT activity in blood serum, mM/h × L	75.8 ± 4.8	54.7 ± 4.7 *	55.5 ± 3.3 *	50.5 ± 3.4 *	62.3 ± 6.3	61.0 ± 2.9 *
De Ritis coefficient	5.01 ± 0.2	5.04 ± 0.2	4.97 ± 0.2	5.68 ± 0.4	5.37 ± 0.3	5.09 ± 0.3
SH-groups in blood serum, µmol/L	14.9 ± 0.5	18.0 ± 1.4	15.7 ± 0.9	16.6 ± 1.8	12.9 ± 1.7	14.9 ± 1.3
Uric acid in blood serum, µmol/L	157.0 ± 11.8	116.6 ± 12.2 *	112.5 ± 11.9 *	119.3 ± 7.9 *	143.3 ± 15.9	124.1 ± 6.7 *
Urea in blood serum, mmol/L	5.4 ± 0.5	5.07 ± 0.6	4.9 ± 0.7	4.6 ± 0.5	5.3 ± 0.5	5.8 ± 0.5
Activity of γ-glutamintransferase in blood serum, nmol/(s × L)	10.6 ± 0.9	8.8 ± 0.8	9.3 ± 1.3	8.1 ± 0.5*	9.05 ± 1.02	12.6 ± 1.3
Creatinine in blood serum, µmol/L	48.4 ± 1.9	44.6 ± 0.9	46.4 ± 0.9	44.3 ± 1.5	44.2 ± 1.1	48.3 ± 1.3
Alkaline phosphatase in blood serum, nmol/(s × L)	110.7 ± 7.9	131.3 ± 8.9	124.8 ± 4.4	115.2 ± 6.2	115.9 ± 8.7	104.3 ± 10.9
Catalase in blood serum, µmol/L	1.6 ± 0.1	0.9 ± 0.2 *	1.1 ± 0.1 *	0.96 ± 0.2 *	1.35 ± 0.1	1.6 ± 0.1
Reduced glutathione in the blood hemolysate, µmol/L	22.6 ± 1.9	18.4 ± 1.1	16.8 ± 1.6 *	22.1 ± 2.6	20.5 ± 1.1	17.8 ± 1.7
Ceruloplasmin in blood serum, mg/%	71.2 ± 2.9	98.3 ± 6.28 *	100.8 ± 6.3 *	101.3 ± 5.0 *	121.6 ± 9.6	75.6 ± 4.5
MDA in blood serum, nmol/L	5.21 ± 0.1	4.5 ± 0.2 *	4.9 ± 0.2	4.55 ± 0.2 *	4.49 ± 0.2	4.92 ± 0.3
Thyrotropic hormone of hypophysis in blood serum, МE/L	1.15 ± 0.2	1.2 ± 0.2	1.7 ± 0.4	1.5 ± 0.3	0.96 ± 0.4	1.0 ± 0.2
Thyroxin in blood serum, pmol/L	6.7 ± 0.6	3.8 ± 0.3 *^,+^	4.8 ± 0.5 *	4.7 ± 0.4 *	4.6 ± 0.4	4.8 ± 0.2 *
Triiodothyronine in blood serum, pmol/L	12.8 ± 0.9	14.8 ± 1.6	13.7 ± 1.0	13.7 ± 0.7	13.2 ± 0.7	11.2 ± 0.6
Diuresis, mL	32.7 ± 1.8	30.0 ± 3.1 ^+^	34.7 ± 3.5 ^+^	17.9 ± 2.9 *	30.2 ± 2.7^+^	31.2 ± 4.5
Urine relative density	1.017 ± 0.001	1.017 ± 0.002 ^+^	1.016 ± 0.001 ^+^	1.023 ± 0.001 *	1.019 ± 0.001 ^+^	1.019 ± 0.001
Protein in urine, g /L	0.13 ± 0.02	0.15 ± 0.01	0.11 ± 0.01 ^+,○^	0.19 ± 0.04	0.16 ± 0.03	0.13 ± 0.01
Urea in urine, mmol/L	146.8 ± 8.4	154.6 ± 8.9	143.3 ± 9.1	183.6 ± 15.3	147.4 ± 8.4	134.2 ± 7.6
Uric acid in urine, µmol/L	91.8 ± 8.9	138.3 ± 11.4 *	98.4 ± 6.0 ^+,○^	166.0 ± 19.1 *	116.6 ± 11.9	89.8 ± 6.2
Creatinine in urine, mmol/L	1.09 ± 0.1	1.4 ± 0.1 *	1.13 ± 0.08 ^+^	1.8 ± 0.2 *	1.2 ± 0.1 ^+^	1.2 ± 0.1
Endogenous creatinine clearance, mL/min	0.76 ± 0.03	0.90 ± 0.03 *^,+^	0.84 ± 0.05	0.66 ± 0.07	0.78 ± 0.04	0.72 ± 0.05
δ–ALA in urine, µmol/L	6.7 ± 1.6	8.27 ± 1.8	11.9 ± 1.8	9.0 ± 1.5	6.2 ± 0.6	8.4 ± 1.3
δ–ALA in urine, µmol/day	0.23 ± 0.07	0.29 ± 0.05	0.34 ± 0.08	0.54 ± 0.13	0.22 ± 0.02 ^+^	0.25 ± 0.08

* statistically significant difference from the control group; ^+^ from the group given NiO-NPs + Mn_3_O_4_-NPs (without the BPC); ^○^ from the group given Mn_3_O_4_-NPs; (*p* < 0.05 by Student’s *t*-test with Bonferroni correction).

It is only in response to the exposure to Mn_3_O_4_-NPs, both alone and in combination with NiO-NPs, that the concentration of uric acid in the urine was substantially and statistically significantly elevated. This effect was the highest in the group exposed to the combination of the NPs (although it was not significantly different from the group exposed to Mn_3_O_4_-NPs alone), while the exposure to NiO-NPs alone did not have any influence on the concentration of uric acid at all. In contrast, the concentration of uric acid in the blood serum was significantly reduced compared with the control values in all groups with the exception of the one exposed to the combination of Mn_3_O_4_-NPs + NiO-NPs with BPC administration.

In the group that was exposed to the same combined impact of Mn_3_O_4_-NPs + NiO-NPs while being administered the BPC, intoxication, as was noted above, did not manifest itself in a statistically significant shift from the control value even if in one index, while in eight of them (average volume of red blood cells, number of leukocytes, bilirubin and albumin contents of the blood serum, diuresis, specific density of the urine, creatinine and δ-ALA contents of the urine) the difference of the group “Mn_3_O_4_-NPs + NiO-NPs + BPC” from the group “Mn_3_O_4_-NPs + NiO-NPs” was statistically significant.

**Figure 2:**
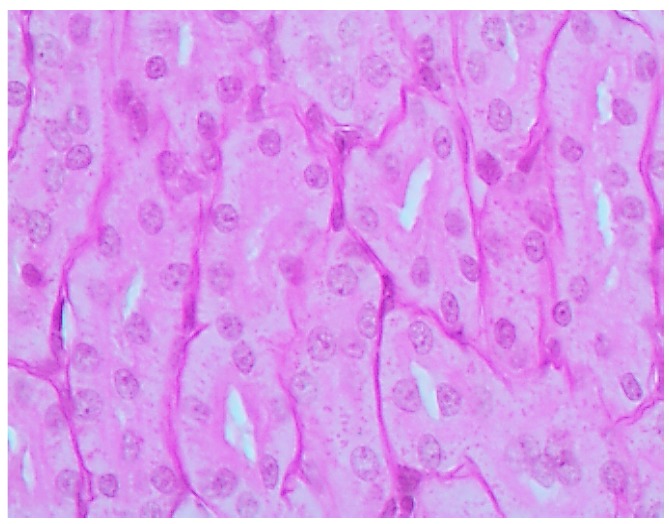
Kidney of a control rat (proximal convoluted tubules with an intact brush border). Periodic Acid Shiff (PAS) stain, magnification ×400.

**Figure 3:**
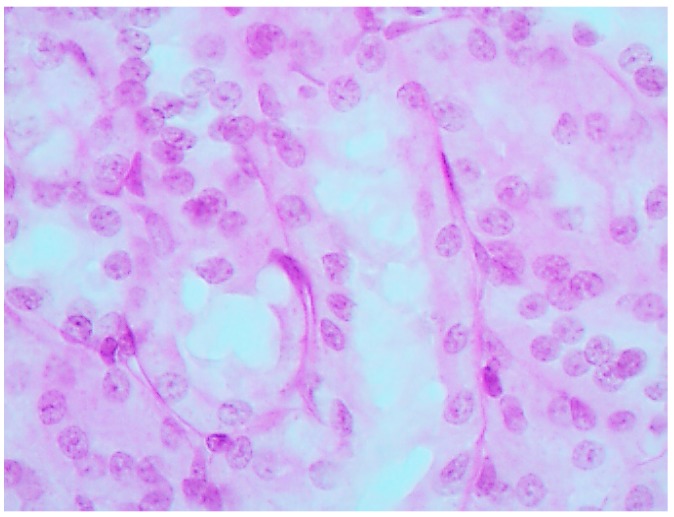
Kidney of a rat exposed to nanoparticles of NiO and Mn_3_O_4_ together. Marked degenerative and necrobiotic changes of tubular epithelial cells up to their disappearance; partial destruction of the brush border. PAS stain, magnification ×400.

**Figure 4:**
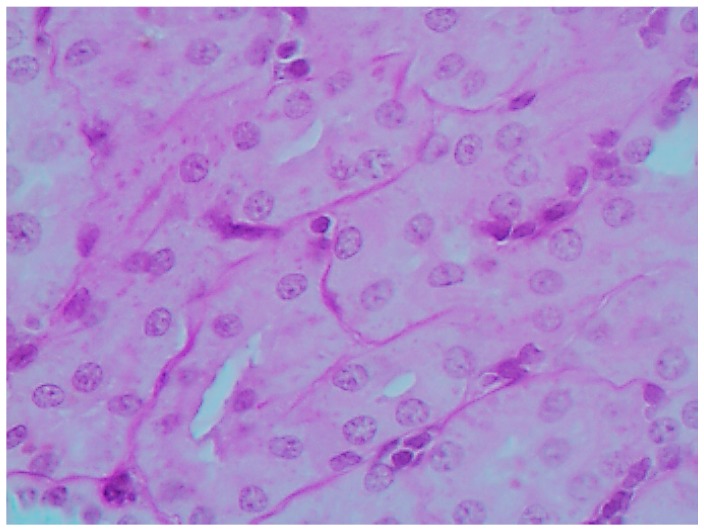
Kidney of a rat exposed to nanoparticles of NiO and Mn_3_O_4_ together against the background BPC administration. Marked alleviation of tubular damage (compare with Figure 2 and Figure 3). PAS stain, magnification ×400.

The histological picture (Figure 2, Figure 3 and Figure 4) and morphometric characteristic (Table 4) of the epithelium in the proximal convoluted tubules provide evidence of marked damage, which is a little more marked in response to NiO-NPs and, particularly, Mn_3_O_4_-NPs + NiO-NPs, than to Mn_3_O_4_-NPs alone, but is absent for the same combined intoxication with the background administration of the BPC.

**Table 4:** Morphometric indices for tubular epithelium damage in the kidneys of rats after repeated intraperitoneal injections of NiO or Mn_3_O_4_ nanoparticles, together or separately, and of their combination with background oral administration of the BPC (X ± s.e.).

Groups of Rats Given	Brush Border Loss (% Lengthwise)	Epithelial Desquamation (% Lengthwise)
Water (control) (4 rats)	5.44 ± 0.9	0.00 ± 0
NiO-NPs (4 rats)	10.3 ± 1.7 *	0.48 ± 0.36
Mn_3_O_4_-NPs (4 rats)	9.02 ± 1.17 *	0.28 ± 0.32 ^+^
NiO-NPs + Mn_3_O_4_-NPs (4 rats)	12.33 ± 2.3 *	2.43 ± 1.0 *
NiO-NPs + Mn_3_O_4_-NPs + BPC (4 rats)	7.08 ± 1.7	0.00 ± 0 ^+^

* statistically significant difference from the control group; ^+^ from the group given NiO-NPs + Mn_3_O_4_-NPs (without the BPC); (*p* < 0.05 by Student’s *t*-test with Bonferroni correction).

The morphometry of the histological liver preparations (Table 5) revealed an increased number of akaryotic hepatocytes in all groups exposed to the NPs. However, this effect was neither high nor statistically significant in the group under combined exposure to NiO-NPs + Mn_3_O_4_-NPs with the BPC administered. Neither significant was the difference between the effects of NiO-NPs and Mn_3_O_4_-NPs, but the combined action of these NPs caused the least increase in the number of akaryotic hepatocytes, being statistically significantly different from the effect of NiO-NPs. The increase in the number of Kupffer cells was, on the contrary, maximal under the combined impact and statistically significant compared not only with the control value but also with the indices for both separate exposures, being absent at all under the effect of NiO-NPs. With BPC being administered, this index was in the group exposed to NiO-NPs + Mn_3_O_4_-NPs not statistically significantly different from the control value and statistically significantly lower than in the group of the same combined exposure without BPC administration. The number of binucleated hepatocytes was considerably reduced only for the effect of NiO-NPs alone.

**Table 5:** Morphometric indices for the state of rat’s liver and spleen after repeated intraperitoneal injections of NiO or Mn_3_O_4_ nanoparticles, together or separately, and of their combination with background oral administration of the BPC (X ± s.e.).

Indices	Groups of Rats (4 Rats)				
	Control	NiO-NPs	Mn_3_O_4_-NPs	NiO-NPs + Mn_3_O_4_-NPs	Nio-NPs + MN_3_O_4_-NPs + BPC
Liver					
Akaryotic hepatocytes per 100 liver cells	2.73 ± 0.28	7.93 ± 0.51 *^,+^	6.03 ± 0.42 *	5.43 ± 0.41 *	3.38 ± 0.34 ^+^
Binucleated hepatocytes per 100 liver cells	17.15 ± 0.66	14.33 ± 0.63 ^+^	18.03 ± 0.60	18.40 ± 0.92	16.20 ± 0.72
Kupffer cells per 100 liver cells	11.25 ± 0.55	10.20 ± 0.63 ^+^	12.75 ± 0.45 *^,+^	16.30 ± 1.22 *	12.2 ± 0.7 ^+^
Spleen					
Red pulp to white pulp ratio	8.3 ± 1.4	4.5 ± 0.9 *	5.2 ± 1.3	3.6 ± 0.4 *	6.2 ± 1.3 ^+^
Number of brown pigment micro aggregates per square of the avtandilov’s grid	0	2.5 ± 0.2 ^○^	6.4 ± 0.3 *^,+^	10.4 ± 0.4 *	5.9 ± 0.2 *^,+^

* statistically significant difference from the control group; ^+^ from the group given NiO-NPs + Mn_3_O_4_-NPs (without the BPC); ^○^ from the group given Mn_3_O_4_-NPs; (*p* < 0.05 by Student’s *t*-test with Bonferroni correction).

In the spleen (Table 5), both types of NPs, particularly Mn_3_O_4_-NPs and, to even greater extent, the combination of Mn_3_O_4_-NPs + NiO-NPs caused a decrease of the red pulp to white pulp area ratio and an increase in the number of brown pigment microaggregates (which could be identified with Perl’s stain as iron-containing). With the BPC being administered, these effects of combined toxic exposure were statistically significantly reduced.

**Table 6:** Morphometric indices for the state of the rat’s brain after repeated intraperitoneal injections of NiO or Mn_3_O_4_ nanoparticles, together or separately, and of their combination with background oral administration of the BPC (X ± s.e.).

Neurons (%)	Control (4 Rats)	Mn_3_O_4_-NPs (4 Rats)	NiO-NPs (4 Rats)	NiO-NPs + Mn_3_O_4_-NPs (4 Rats)	NiO-NPs + Mn_3_O_4_-NPs and BPC (4 Rats)
Nucleus caudatus					
Without a nucleolus	30.50 ± 2.77	69.90 ± 1.79 *^,+^	47.36 ± 2.45 *^,+^	60.30 ± 2.26 *	37.15 ± 2.89 ^+^
With a distinct centrally located nucleolus	25.12 ± 1.16	9.33 ± 0.90 *^,+^	17.00 ± 1.04 *^,+^	12.35 ± 0.95 *	23.28 ± 1.09 ^+^
Hippocampus (CA 1)					
Without a nucleolus	30.50 ± 2.30	70.45 ± 2.31 *	35.8 ± 2.21 ^+^	70.40 ± 3.75 *	41.30 ± 2.14 *^,+^
With a distinct centrally located nucleolus	46.4 ± 2.92	13.4 ± 1.51 *	31.6 ± 1.75 *^,+^	11.0 ± 1.13 *	30.5 ± 1.96 *^,+^

* statistically significant difference from the control group; ^+^ from the group given NiO-NPs + Mn_3_O_4_-NPs (without the BPC); (*p* < 0.05 by Student’s *t*-test with Bonferroni correction).

**Figure 5:**
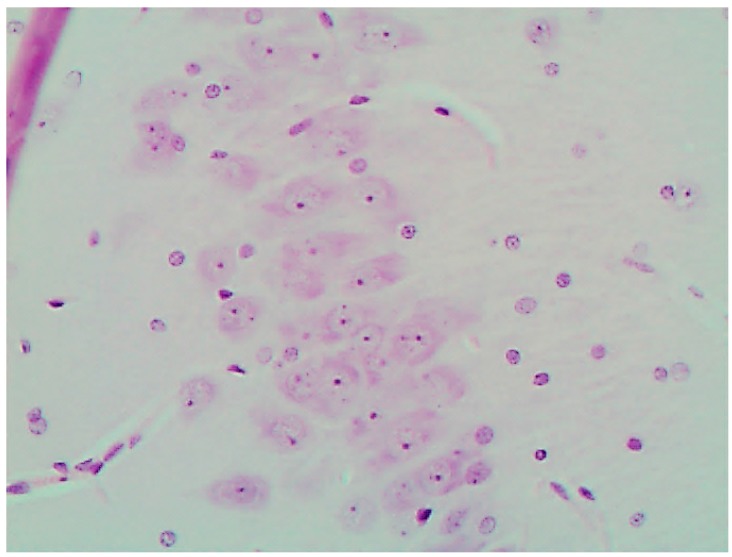
The brain of a control rat, hippocampus (CA1). Hematoxylin and eosin stain, magnification ×400. The neuron nuclei are predominantly spherical with well-visible eosinophilic granulosity, and notable nucleoli in the center.

**Figure 6:**
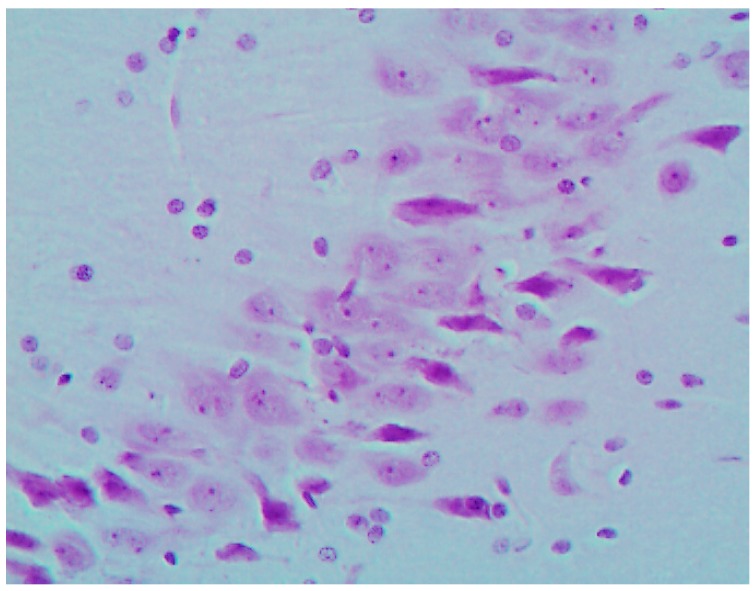
The brain of a rat exposed to nanoparticles of NiO and Mn_3_O_4_ together; hippocampus (CA1). Hematoxylin and eosin stain, magnification ×400. A lot of neurons with marked degenerative changes or pycnosis; in some nuclei the nucleolus is displaced or absent.

**Figure 7:**
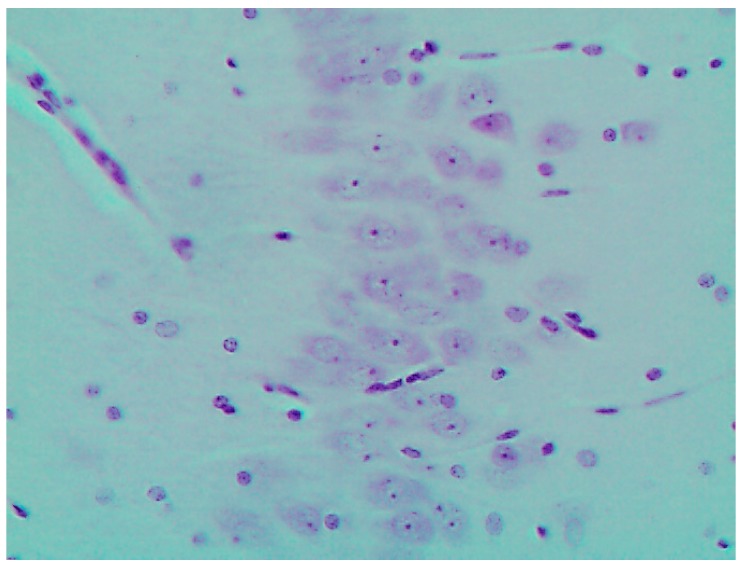
The brain of a rat exposed to nanoparticles of NiO and Mn_3_O_4_ together against the background BPC administration; hippocampus (CA1). Hematoxylin and eosin stain, magnification ×400. Marked alleviation of neuronal damage (compare with Figure 5 and Figure 6).

The most prominent and similar pathological changes of brain structures were revealed in the area of caudate nucleus and in the CA1 area of the hippocampus; as examples, we give microscopic pictures of the latter in a control rat (Figure 5) and in one exposed to Mn_3_O_4_-NPs + NiO-NPs (Figure 6) Both morphometric indices of the brain’s histological picture used in our study, namely, counts of neurons with completely absent nucleolus and of those with a distinct nucleolus located in the nucleus centre show substantial damage to these structures of the brain mostly in the rats exposed to either Mn_3_O_4_-NPs or Mn_3_O_4_-NPs + NiO-NPs. This adverse effect is almost absent in rats exposed to the same toxic combination with the background BPC administration (Table 6 and Figure 7).

The coefficient of genomic DNA fragmentation in the blood nucleated cells (Table 7) proved to be statistically significantly and virtually equally elevated in the rats exposed to all the NPs tested, but the background BPC administration significantly reduced the effect of the combined toxic exposure while the BPC alone did not produce any significant influence on the control value.

**Table 7:** The coefficient of genomic DNA fragmentation in the blood nucleated cells of rats after repeated intraperitoneal injections of NiO or Mn_3_O_4_ nanoparticles, together or separately, and of their combination with background oral administration of the BPC (X ± s.e.).

Control (6 Rats)	Mn_3_O_4_-NPs (6 Rats)	NiO-NPs (6 Rats)	NiO-NPs + Mn_3_O_4_-NPs (6 Rats)	NiO-NPs + Mn_3_O_4_-NPs and BPC (6 Rats)	BPC (6 Rats)
0.42 ± 0.00	0.51 ± 0.01 *	0.51 ± 0.01 *	0.50 ± 0.01 *	0.45 ± 0.01 *^,+^	0.41 ± 0.01

* statistically significant difference from the control group; ^+^ from the group given NiO-NPs + Mn_3_O_4_-NPs (without the BPC); (*p* < 0.05 by Student’s *t*-test with Bonferroni correction).

## 3. Discussion

Using intraperitoneal injections for modeling systemic intoxication, which in real conditions can be induced by long term inhalation exposure of workers needs some justification. It is well known that “nanoparticles deposit with high efficiency in the entire respiratory tract, from the head airways to the alveoli, due to diffusion” [26]. For instance, the widely used Human Respiratory Tract Model (HRTM) of the International Commission of Radiological Protection (ICRP) [27,28] predicts 100% total deposition of 0.001 μm (*i.e.,* 1 nm) and ~90% for 0.01 μm (*i.e.*, 10 nm) particles for a normal adult mouth breathing male human subject. However, there are many anatomical, functional and aerodynamic differences between humans and rodents which make one assume interspecies distinctions in regional particle deposition and thus in the kinetics of their elimination from the airways to the GIT and/or absorption. No wonder that the authors of a comprehensive review of nano-toxicological assessment techniques [29] maintain that “rodents, the commonly used species for toxicology testing, are obligatory nose breathers and, therefore, not representative models for human respiratory inhalation exposure”. In other words, NP inhalation by laboratory rodents is not as ideal a model of real human exposures as it is often deemed to be.

The intraperitoneal model permits one to circumvent these interspecies differences of inhaled NP deposition and is adequate enough when one wants to look into body distribution and elimination of NPs, and for organism’s reactions to NPs after they have penetrated into the blood from a primary deposit. Like any model (always a necessary simplification of a complicated system deliberately omitting some sub-systems and some material or informational flows and feedbacks), it has both drawbacks and virtues. Among the latter, one should take into consideration that dosing by injection is much more accurate and reliable as compared with the more “natural” experimental methods. This reason is crucial for experiments of comparative design like ours. Intraperitoneal modeling of subchronic intoxications is well known and recognized in general experimental toxicology. Moreover, it was used not only by us [1,2,4,6] but also by other researchers [30,31] just in experimental nano-toxicological studies.

In this Section, we might first have discussed all the detected toxic effects of the studied NPs and then pass on to discussing the preventative efficacy of the bio-protective complex (BPC). However, although the toxicity attenuating action of this BPC is important as such, especially from the practical point of view, it is also a useful research tool. It helps to additionally verify the causality of this or that difference between the group exposed to the toxic impact and the control group, and to demonstrate that a difference is indeed due to this impact rather than to sampling errors. Despite the importance of the widely used statistical methods for null hypothesis testing, they always leave room for doubt. Meanwhile, there is a long established diagnostic approach in clinical medicine known as making a diagnosis *ex juvantibus* (that is making an inference about disease causation from its beneficial response to a specific treatment), which can be used in experimental toxicology as well. This approach was used by us for presenting our research on CuO-NPs toxicity [4], but it is not really quite new. For instance, it is fairly common to prove the mechanistic role of oxidative stress under different toxic impacts *in vitro* by reducing it with the help of antioxidants and free radical scavengers.

For these reasons, we propose to discuss the toxic outcomes in parallel with the discussion of changes in them brought about by the background administration of anti-toxic bioprotectors.

The results described in the previous Section provide evidence, first of all, that the subchronic IP exposure of rats to NPs of both metal oxides in the doses used caused the development of a moderately pronounced intoxication which did not lead to the death of the animals or even to a retarded weight gain. Nevertheless, this intoxication was clearly manifest not only through adverse changes in a great number of functional indices of the organism’s status but even through substantial morphological damage to the organs studied.

When comparing the effects of NiO-NPs and Mn_3_O_4_-NPs, noteworthy is the prevailing similarity of the type of changes, tending, however, to be more pronounced under the effect of Mn_3_O_4_-NPs for the majority of the indices.

At the same time, the reduction of the kidney mass and morphometric indices of nephrotoxicity (Table 4) were somewhat more marked in response to the action of NiO-NPs, which is likely to be associated with the predominantly renal route of nickel excretion (in contrast to the predominantly hepato-biliary elimination of manganese). Indeed, the Thus, 24 h urinary excretion of nickel under exposure to NiO-NPs (Table 2) was 13 times higher than the same in control rats and 632 times higher than the respective value for manganese both in controls and under exposure to Mn_3_O_4_-NPs. Given such significant dependence of the chemical stress endured by kidneys in the process of these metals’ elimination on the kind of NPs rats were exposed to, one might predict even much more pronounced difference between indices of their nephrotoxicity than really observed.

The functional and biochemical data summarized in Table 3 point to the protective efficacy of the BPC not only in relation to the changes in the eight effects listed in the previous Section, which proved to be statistically significantly weaker in the case of combined intoxication against the background BPC administration than for the same exposure without such bioprotection. An additional criterion of this efficacy is the fact that a value was no longer statistically significantly different from the control one for not only these but also a great number of other indices.

Moreover, analysis based on a linear discriminant function with stepwise variable selection enabled us to create, using a set of seven indices (out of the ones that did not differ statistically significantly in pairwise comparison) [32], a decision rule that permitted to refer reliably (with from 75% to 100% correct classifications) an observation to one of the groups under comparison: controls, exposed to the combination of particles, or exposed to the same combination with background BPC administration. The squared Mahalanobis distance (which can be regarded as a measure of dissimilarity between groups) proved to be 10.1 between the “Controls” and the “Combined exposure without BPC”, and only 4.6 between the “Controls” and the “Combined exposure with the administration of the BPC”, being equal to 6.6 between the two exposed groups. In other words, even this small set of seemingly insignificant indices suggests that the group exposed to the NPs with the BPC being administered shifted closer to the control group, demonstrating less difference from the latter than from the group exposed to the NPs without BPC administration.

Comparison of functional effects under separate and combined exposures to NiO-NPs and Mn_3_O_4_-NPs reveals their additivity or even synergism judging by some of indices but subadditivity judging by the others. Outside the nanotoxicological agenda, such complex characterization of different metals combined toxicity seems to be a general rule [3] but within it, this question is considered for the first time. Therefore, for an in-depth understanding of the type of combined toxicity of the NPs based on the above-mentioned data and on those discussed below, it was necessary to rely on its mathematical modeling which is considered by us elsewhere.

It is worth specially noting that only exposures to Mn_3_O_4_-NPs, both alone and in combination with NiO-NPs, were associated with a substantially increased concentration of uric acid in the urine. The essential role of uric acid as a component of the organism’s antioxidant system is well known [33]. An experimental intoxication of rats with manganese demonstrated an increased uric acid content of the striatum, presumably associated with the activation of xanthine oxidase [34]. It is quite probable, however, that the effect of manganese on the uric acid balance in the organism is dependent on the level of exposure and/or the phase of intoxication. Indeed, the workers who were exposed to manganese for a long time were found to have a lowered, rather than elevated, concentration of uric acid in the urine in comparison with those who were not so exposed [35]. Also, it should not go unmentioned that, in our experiment, the concentration of uric acid in the blood serum of the rats was, unlike that in the urine, reduced significantly in equal extent in response to Mn_3_O_4_-NPs and NiO-NPs. Comparing this fact with the decrease in the blood serum concentrations of reduced glutathione and two enzymes (catalase and, to a lesser degree, γ-glutamintransferase), also participating in antioxidant protection, we can assume that all these changes reflect an elevated expense of the antioxidant system’s reserves under the oxidative stress caused by both nano-metals. Although we find it difficult to explain the obvious inconsistency between the shifts of the uric acid content in the urine and blood, the association of both shifts with the intoxication is again corroborated by their absence in the group exposed to the combination of Mn_3_O_4_-NPs and NiO-NPs with the background antitoxic bio-protection.

There is no serious doubt as to the specificity of the damaging action of manganese oxide NPs on the neurons of the striatum (in particular, of the caudate nucleus) and the hippocampus. This interpretation is highly plausible considering the well-known key role of the syndrome resembling Parkinson’s disease in the clinical presentation of chronic manganese intoxication in humans. For this reason, we attach special significance to the changes in neurons in these areas of the brain that we found in the rats exposed to Mn_3_O_4_-NPs alone or in combination with NiO-NPs (Figure 5, Figure 6 and Figure 7, Table 6).

Specifically, our microscopic study revealed clear signs of damage to the neuron bodies in both the caudate nucleus and the CA1 area of the hippocampus. In particular, we observed poor staining of the eosinophilic granulosity of the nuclei, disappearance of the nuclear membrane, and pycnosis of the nucleolus, which was often displaced towards the periphery of the nucleus or was absent. Whether these features may be interpreted as light microscopy signs of neuronal apoptosis or of other types of neuron’s toxic damage is difficult to decide without special tests (e.g., an immuno-histochemical detection of caspase 3) but we believe that such an interpretation is plausible enough.

For these brain structures morphometry, we used the disappearance of the nucleolus as the most accurate and unbiased index and, on the other hand, a virtually normal appearance and location of the nucleolus. Both indices testified concurrently to: (a) high neurotoxicity of Mn_3_O_4_-NPs and, to the same degree, of the combination of Mn_3_O_4_-NPs + NiO-NPs; (b) much lower, if any, neurotoxicity of NiO-NPs acting separately; (c) dramatic alleviation of this crucial effect of combined neurotoxicity by background BPC administration.

In the liver, the most remarkable intergroup difference was in the number of Kupffer cells (*i.e.*, activated resident macrophages of this organ) which was statistically significantly increased only under the exposure to Mn_3_O_4_-NPs, both alone and combined with NiO-NPs. We had also observed this effect under subchronic IP exposures to iron oxide NPs [2], metallic silver, metallic gold [6], and copper oxide [4].

This activation of tissue macrophages may be related not only to the capturing of particles by them from the bloodstream. Indeed, judging from the EPR spectroscopy results, the NiO-NPs content of the liver was higher as compared with that for Mn_3_O_4_-NPs while only the latter induced a statistically significant increase in the Kupffer cells count. As is well known, however, resident macrophages play multiple roles in the process of apoptosis of an organ’s functional cells, engulfing, in particular, apoptotic bodies [36]. In the liver, it is just the Kupffer cells that play this important role [37]. Thus, Kupffer cell activation might serve as an indirect indicator of the metallic NPs’ hepatocytotoxic effect, which is indeed known to be associated with apoptosis [38]. The alleviation of this effect of NPs by background administration of bio-protectors reducing their toxicity (demonstrated by us in experiments with Ag-NPs and CuO-NPs as well) provides evidence in favor of such interpretation. We should qualify it, however, assuming that hepatocyte’s death under the impact of the studied NPs might be not only of the apoptotic but also of the necrobiotic kind as is evidenced by the statistically significant increase in the proportion of akaryotic cells (Table 5). Moreover, this index of NP-induced necrosis of hepatocytes was the highest under the exposure to NiO-NPs, while it was just in this group that we did not observe any increase in the Kupffer cell count. This seeming discrepancy of the two effects under discussion might be a circumstantial evidence in favor of our assumption that the mechanisms of hepatocyte killing by Mn_3_O_4_-NPs and by NiO-NPs are somewhat different.

It is of interest as well that the combined effect of these NPs on the Kupffer cell count is explicitly superadditive while that on akaryotic hepatocytes is rather subadditive. Anyway, both hepatic effects under consideration were reduced down to a statistically non-significant level with background BPC administration (Table 5).

This attenuating influence of the BPC on the morphometric indices of liver damage can be juxtaposed with a reduction of the burden on the organ with at least one of the toxic metals (nickel), including in the form of NiO-NPs (Table 1, Figure 1). In the spleen too, a similar beneficial toxicokinetic effect of the BPC might be one of possible causes of the attenuation of this organ’s responses to the combined toxic impact (Table 5). In particular, the BPC decreased significantly accumulation of an iron-containing pigment (presumably, hemosiderin) probably associated with the capturing by the activated resident splenic macrophages of more rapidly aging erythrocytes from the bloodstream and with their enhanced disposal. This hypothesis may be substantiated by the fact that the BPC attenuated also the hemolytic anemia caused by combined toxic exposure (Table 3). As to a seemingly paradoxical decrease, under the toxic exposure, of the red pulp to the white pulp planimetric ratio, it was due not to decrease in the former but to increase in the latter, evidently, as a reaction of lymphoid tissue to metals toxicity. This hyperplasia of the white pulp was reflected by the increase in the spleen mass (Table 3) Anyway, a significant attenuation of all these shift against the background BPC administration suggests again that they were not chance findings but effects of the NPs toxicity.

It should be noted, however, that we discovered but few such explicitly beneficial toxicokinetic effects of the BPC, and we have not found them even in the organs for which the attenuation of toxicodynamic effects under the influence of the same BPC causes no doubts. It is especially noteworthy in this respect that under combined toxic exposure the action of the BPC did not decrease (rather, increased a little) the manganese content of the rat’s brain but lowered sharply and statistically significantly the nickel content. Meanwhile, as follows from Table 7, the BPC substantially reduced morphological damage to the target brain structures of the rats under combined exposure, which damage in this experiment was most probably associated more with the neurotoxicity of manganese than with that of nickel. It should be noted, in this context, that we observed similar damage in the caudate nucleus under subchronic exposure to CuO-NPs [4] and that in that experiment as well a substantial attenuation of the effect under the influence of a BPC was not accompanied by a reduction in the copper content of the brain as a whole.

We believe that this and other seeming contradictions between a high protective efficacy of the BPC in relation to the effects of toxic action and low (and even paradoxical) dependence of the accumulation of toxic metals in the organs on the same BPC point to the fact that beneficial toxicodynamic effects of bioprotectors are associated with various mechanisms (partly touched upon above in Section 2 when justifying the choice of bioprotectors), among which mechanisms a reduction in the tissue dose of metals is far from necessary being the key one.

Moreover, we found quite a long ago that in various metal intoxications this reduction is often not only, and not so much, the cause of attenuation of the toxic effect as the latter’s result [24]. In the present case, for example, the BPC could reduce the body burden and thus the retention of nickel in different organs because of the enhanced excretion of this metal with urine which, in is turn, might be due to the alleviation of the kidney damage.

As is it has been already mentioned above, kidneys are the main organ of excretion for nickel (unlike manganese) and, in our experiment, the exposure to NiO-NPs caused a sharp increase in renal excretion of nickel while a similar exposure to Mn_3_O_4_-NPs did not result in a measurable increase in the excretion of manganese. At the same time, nickel excretion was significantly lower under exposure to the combination of the metals than to NiO-NPs alone, while manganese excretion, on the contrary, was significantly higher than under exposure to Mn_3_O_4_-NPs alone. These seeming contradictions can be hypothetically explained as follows. Toxic damage to the kidneys, greatest just under combined exposure, presumably reduced their ability to eliminate both metals. However, in relation to the renal excretion of manganese, another mechanism seems to have prevailed, the one associated with predominantly erythrocyte-bound transport of this metal by the blood. Admittedly, the accelerated ageing of erythrocytes under the effect of nickel [39] leading to their more extensive destruction, redistributes part of the manganese into the plasma, thus enabling the kidneys to eliminate it. Circumstantial evidence for the consistency of these hypotheses is that both seemingly contra-directional toxicokinetic effects of combined exposure to NiO-NPs + Mn_3_O_4_-NPs were attenuated by the BPC, which also reduced both the damage to the kidneys (Table 4) and the decrease in the erythrocytes count (Table 3).

Earlier we demonstrated the absence of an unequivocal and easily interpreted conformity between toxicokinetic and toxicodynamic indices also for the effects of combined impact of two or three metals in the form of water-soluble salts, including the combination of KMnO_4_ + NiCl_2_ [22]. Such interpretation is even more difficult under a combined action of metallic nanoparticles because the toxicokinetics of the same metals in this case is controlled by considerably distinct toxicokinetic mechanisms for persisting NPs and for metal ions released by NPs’ dissolution, the balance of these mechanisms being different for NPs of different solubility. The outcome is such paradox as the substantial increase in the retention of total nickel in the liver due to the accompanying impact of manganese, (Table 1), and, in the same time, a decrease in the retention of nickel in the form of NiO-NPs in organs where its amount would be sufficient for measurement, *i.e.*, again in the liver and in the spleen (Figure 1).

The seemingly odd circumstance that we failed to find any accumulation of Mn_3_O_4_-NPs even in these organs as well as the fact that in all organs studied the total manganese accumulation was lower than total nickel accumulation can be explained by faster dissolution *in vivo* of manganese oxide nanoparticles compared with nickel oxide ones. This hypothesis was supported, in an additional model *in vitro* experiment, by a comparative assessment of the solubility of Mn_3_O_4_-NPs and NiO-NPs in sterile bovine blood serum. After 24 h dissolution, the serum concentration of Mn_3_O_4_-NPs reduced 5 times while the concentration of NiO-NPs diminished by 7% only. The decay of EPR signal kinetics was fitted: for Mn_3_O_4_-NPs_,_ by a two-exponential function with time constants τ_1_ = 20 ± 3 min and τ_2_ = 425 ± 100 min, while for NiO-NPs by a single exponential function with τ = 405 ± 70 min.

Genotoxicity of many metallic nanoparticles was demonstrated by a lot of researchers although mostly in *in vitro* experiments on stable cell lines. In our previous studies we were able to demonstrate, with the help of the RAPD test, a significant *in vivo* damage to the genomic DNA of target organs’ cells and of nucleated blood cells under subchronic exposures to silver NPs, gold NPs [6] and copper oxide NPs [4]. In the experiments with Ag-NPs and CuO-NPs we tested in parallel the same DNA-damaging effect in rats exposed to these NPs and given BPCs having a composition similar to that used in the present study (although differing in some details). We found that in both cases this effect was significantly alleviated. This experience permits us, as we believe, to consider the RAPD-test that in this study was performed only on blood cells (Table 7) as a “monitor” of the *in vivo* genotoxicity on the organism level of manganese and nickel NPs acting separately or in combination and of its alleviation by the BPC.

By way of concluding the discussion of our results, we cannot avoid considering the issue of the statistically significant shifts in comparison with the control values that were observed for 6 functional indices in the group receiving BPC alone, namely: increase in the average volume of red blood cells, decrease in SDH activity in lymphocytes, and the concentrations of AST, ALT, uric acid and thyroxin in blood serum. The physiological or diagnostic importance of these shifts is hardly significant not only because their absolute values are not high but also because they appear to be occasional rather than systemic. Thus, the BPC did not change any other index of the red blood in non-exposed rats except for a small increase in the average volume of RBC; nor were reduced the levels of other blood serum enzymes but the just listed.

However, it is even more important that comparison of the group administered the BPC in parallel to combined toxic exposure with the group exposed to the same combination without BPC administration shows that the discussed effects of the latter are essentially different. Indeed, such comparison reveals no influence of the BPC on the thyroxin content of the blood serum, while for the other above-mentioned five indices it had the opposite sign, bringing them closer to the control values, *i.e.*, being explicitly beneficial.

Thus, the expectation stated in Section 2 when justifying the composition and doses of the bioprotectors tested, namely that an additional intake of some biologically active micronutrients over the balanced food might be excessive for the organism living under normal conditions but is quite justified in the conditions of toxic stress caused by chronic exposure to the nanoparticles studied, has been confirmed. Aiming at an eventual controlled trial of this BPC on volunteers just in the conditions of such exposure [24], we believe there are all grounds to expect a high protective efficacy and not to be afraid of any side-effects of this BPC.

## 4. Experimental Section

The experiment was carried out on outbred white female rats from our own breeding colony with the initial body weight of 150 to 220 g, with minimum 12 animals in different exposed and control groups (the actual numbers of rats used for this or that post-exposure investigation are given in Table 1, Table 2, Table 3, Table 4, Table 5, Table 6 and Table 7). Rats were housed in conventional conditions, breathed unfiltered air, and were fed standard balanced food. The experiments were planned and implemented in accordance with the “International guiding principles for biomedical research involving animals” developed by the Council for International Organizations of Medical Sciences (1985) and were approved by the Ethics Committee of the Ekaterinburg Medical Research Center Medical for Prophylaxis and Health Protection in Industrial Workers.

For this experiment, we prepared suspensions of metal oxide nanoparticles by laser ablation of pure metal targets in water. A plate of pure nickel or manganese with a metal content of 99.9% was placed on the bottom of a shallow tray with deionized water. Metal ablation was performed using an Fmark-20RL laser material processing system (Laser Technology Center, St. Petersburg, Russia), based on ytterbium-doped pulsed fiber laser (pulse length 100 ns, repetition rate 21 kHz, wavelength 1064 nm). The energy density was about 80 J/cm^2^. The target was irradiated in scanning mode with the rate of the laser spot at 270 mm/s. At the beginning, seven scanning cycles were used for preparation of the target surface.

A scanning electron microscope (SEM), CrossBeam Workstation Auriga (Carl Zeiss, Jena, Germany), was used for the visualization of the nanoparticles. A Raman confocal microscope, Alpha 300 AR (WiTec, Ulm, Germany), was used for analysis of the NP composition.

**Figure 8:**
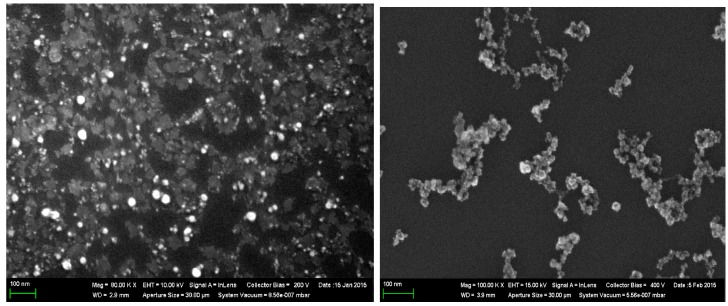
SEM images of NiO (**left**) and Mn_3_O_4_ (**right**) nanoparticles in suspension obtained by scanning electron microscopy.

**Figure 9:**
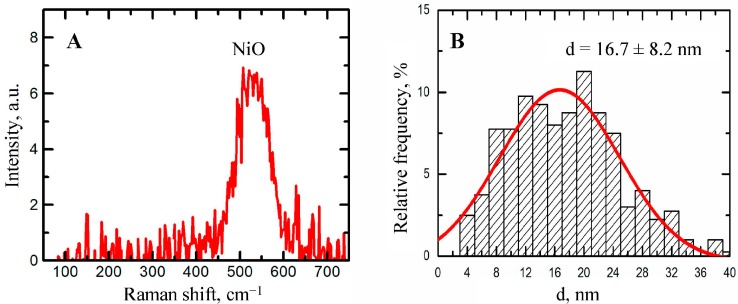
Characteristics of nickel oxide NPs produced by laser ablation. (**A**) Raman spectrum; (**B**) size distribution function obtained by statistical processing of SEM NPs images.

**Figure 10:**
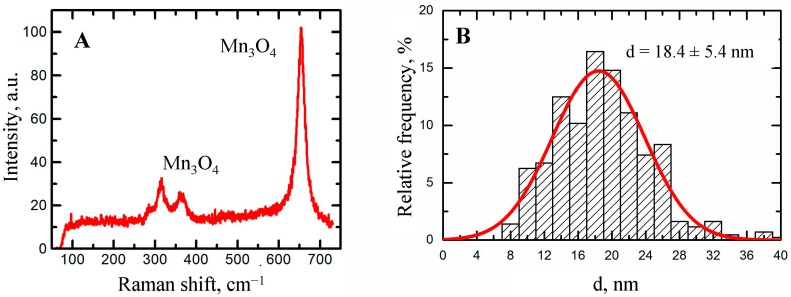
Characteristics of manganese oxide nanoparticles produced by laser ablation. (**A**) Raman spectrum; (**B**) size distribution function obtained by statistical processing of SEM images of NPs.

The concentration was increased to 0.5 mg/mL by partial evaporation of the primary suspensions for 5 h at 50 °C. The nanoparticles in both suspensions were of spherical shape (Figure 8). Their chemical composition was revealed by Raman Spectroscopy (Figure 9A and Figure 10A). The average particle diameter (±s.d.) obtaineda by statistical processing of hundreds of scanning electron microscopy (SEM) images was 16.7 ± 8.2 nm for NiO-NPs and 18.4 ± 5.4 nm for Mn_3_O_4_-NPs. The distribution functions (Figure 9B and Figure 10B) were symmetrical in both cases.

The absence of noticeable changes in the zeta potential as well as in the shape and position of the plasmon resonance peak 30 days after suspension preparation confirmed the high stability of the NiO-NPs suspension. The Mn_3_O_4_-NPs suspension was less stable, but disaggregation of nanoparticles was easily achieved by a short-run ultrasonication just prior to the injection. No chemical stabilizers were added to either of the suspensions.

Each nanomaterial thus prepared was administered to the rats intraperitoneally (IP) 3 times a week (up to 18 injections) at a dose of 0.5 mg per rat (*i.e.*, about 2.5 mg/kg) in 1 mL of the suspension. To avoid direct interactions between chemically different NPs resulting in their fast aggregation, the NiO-NPs and Mn_3_O_4_-NPs suspensions were drawn into different syringes and injected separately to the rats of the combined exposure group, one after another, at an interval of about 1 min. The rats of the groups exposed to NPs of either NiO-NPs or Mn_3_O_4_-NPs alone were additionally injected with the same volume of sterile deionized water (from the batch used for preparing the suspensions) by the same route, while the rats of the control group received only IP injections of this water.

A special group of rats was injected with the same dosage of (NiO-NPs + Mn_3_O_4_-NPs) but with background administration of a bio-protective complex (BPC) described below, and still another group was given the same BPC plus IP injections of water.

Immediately after the end of the exposure period, the following procedures were performed for all rats:

- Weighing.
- Estimation of the CNS ability for temporal summation of sub-threshold impulses—A variant of the withdrawal reflex and its facilitation by repeated electrical stimulations in an intact, conscious rat.
- Recording of the number of head-dips into the holes of a hole-board (which is a simple but informative index of exploratory activity frequently used for studying the behavioral effects of toxicants and drugs).
- Collection of daily urine for analysis of its output (diuresis), specific gravity (density), protein, total coproporhyrin, δ-aminolevulinic acid (δ-ALA), urea, uric acid, creatinine, and Ni and Mn contents.
- Then the rats were killed by decapitation and blood was collected by exsanguination. The liver, spleen, kidneys, and brain were weighed. The biochemical indices determined from the blood included reduced glutathione (GSH), total serum protein, albumin, globulin, bilirubin, ceruloplasmin, malonyldialdehyde (MDA), alkaline phosphatase, alanine- and asparate-transaminases (ALT, AST), catalase, gamma glutamyl transferase, SH-groups, urea, uric acid, creatinine, thyrotropic hormone of hypophysis, thyroxin, and triiodothyronine. For determining hemoglobin content, hematocrit, mean erythrocyte volume and for counting RBC, WBS and thrombocytes we used the MYTHIC-18 auto-hematology analyzer. Reticulocytes percentage was counted using the routine technique. Cytochemical determination of succinate dehydrogenase (SDH) activity in lymphocytes was based on the reduction of nitrotetrazolium violet to formasan, the number of granules of which in a cell is counted under immersion microscopy.

All the clinical laboratory tests on blood and urine bar specially stipulated were performed using well-known techniques described in many manuals [40].

The statistical significance of the differences between the group arithmetic mean values was estimated using Student’s *t* test with Bonferroni correction for multiple comparisons, but the tables of results below do not specify it for the groups, comparing which is senseless at all. Specifically, we do not compare the group exposed to NiO-NPs + Mn_3_O_4_-NPs + the BPC with those exposed to either NiO-NPs or Mn_3_O_4_-NPs without the BPC; neither do we compare all three groups exposed to these NPs (separately or in combination) with the one given the BPC without any NPs.

The total concentrations of the metals (Ni and Mn) in the samples of the liver, kidneys, spleen and brain were measured by the atomic emission spectroscopy (AES) method using an atomic emission spectrometer with inductively coupled plasma iCAP 6500 Duo (Thermo Scientific, Waltham, MA, USA). Samples of the freeze-dried homogenized tissue were subjected to acid ignition with the help of a MARS 5 microwave accelerated reaction system (Matthews, NC, USA). The NiO-NPs and Mn_3_O_4_-NPs NP contents of the samples of the same organs were revealed by the electron paramagnetic resonance (EPR) method using an electron paramagnetic resonance spectrometer EMX Plus (Bruker, Karlsruhe, Germany). The measurements of the freeze-dried homogenized tissue were done at room temperature.

Liver, spleen, kidney, and brain tissue sections were prepared from four rats from each treated and control group for histological examination by the hematoxilin and eosin stain and, when necessary, PAS, Nissl or Perl’s stain. For morphometric characterization of these tissues we used the Avtandilov’s planimetric ocular grid and the image recognition programmed system CellSens (Olympus, Hamburg, Germany) [41].

### 4.1. Testing of the in Vivo Genotoxicity with the Random Amplification of Polymorphic DNA (RAPD) Test on Blood Nucleated Cells

Totally, we analyzed 66 blood samples, each sample in three replications. The samples were collected into special vessels cooled to −80 °C. These were then promptly delivered in cryo-containers to a specialized laboratory. To isolate DNA from the cells, we used a GenElute (Sigma, St. Louis, MO, USA) set of reagents in accordance with the manufacturer’s guidelines for use. The DNA content of the samples was determined spectrophotometrically (Ultraspec 1100 pro; Amersham Biosciences Ltd.: Amersham, UK); then they were frozen and stored at −84 °C in a kelvinator (Sanyo Electric Co., Ltd.: Moriguchi, Japan) till the beginning of the RAPD (Random Amplified Polymorphic DNA) test performed as described by us earlier [42]. The method is based on the fact that, unlike a fragmented DNA, which, in the agarose gel electrophoresis, forms the so-called comet tail, a non-fragmented DNA has a very low degree of migration and virtually stays in the same place (comet head), the degree of migration being directly related to the degree of DNA fragmentation. DNA amplification was carried out using specific primers and tritiated nucleotides. To characterize the degree of damage to DNA we used the “coefficient of fragmentation”, *i.e.*, the ratio of total radioactivity of all tail fractions to that of the head.

### 4.2. Choice of Bioprotectors

Based on the literature dealing with the mechanisms of toxic action of Ni and Mn and on our experience in the successful testing of various bioprotectors against other intoxications [24], including those caused by nano-Ag [6] and nano-CuO [4], we chose the following substances for estimating their possible protective action against the combined subchronic intoxication with NiO-NPs and Mn_3_O_4_-NPs:

(1) Glutamate as an effective cell membrane stabilizer acting through the intensification of ATP synthesis under exposure to the damaging action of various cytotoxic particles [6,43,44,45] and, at the same time, as one of the precursors of glutathione, which is a powerful cell protector against the oxidative stress, the latter being one of the key mechanisms of virtually all metallic NPs’ cytotoxicity and genotoxicity [5]. In addition to these non-specific and almost universal bio-protective effects of glutamate, we believed that its administration might specifically increase resistance to manganese neurotoxicity due to glutamate’s major role in transmitting excitatory signals in the mammalian central nervous system and thus involvement in most aspects of normal brain functioning. Indeed, although the mechanisms of manganese neurotoxicity are yet to be elucidated, it is widely maintained that manganese impairs the expression and function of the main glutamate transporters in astrocytes [46,47]. We therefore supposed that additional glutamate supply to the cells would partly compensate for this adverse effect of manganese toxicity.

(2) The other two glutathione precursors: glycine and cysteine (the latter in a highly active and metabolically well available form of *N*-acetylcysteine), taking into consideration both the general important role played by the oxidative stress as a mechanism of metallic NPs cytotoxicity and the experimental data demonstrating that glutathione deficiency potentiates manganese toxicity in the rat striatum and brainstem [48].

(3) Other components of the organism’s anti-oxidant system (vitamins A, E, and C, and selenium).

(4) ω-3 polyunsaturated fatty acids, whose intracellular derivatives are eicosanoids that activate DNA replication and thus play an important part in its repair, which was demonstrated by us earlier under exposure to various genotoxic agents (e.g., [4,6,42]).

(5) Iodine, taking into consideration complex disturbances of the thyroid function caused by manganese intoxication [49].

(6) Pectin enterosorbent as an agent that prevents the re-absorption of toxic metals excreted into the intestines with bile (which, again, is of special importance for manganese as it is excreted predominantly by this route).

The doses and administration methods of these bio-protectors are given in Table 8.

**Table 8:** Doses and the mode of administration of the bioprotectors tested in our experiment.

Bioprotectors	Estimated Dosage and the Mode of Administration
Apple pectin	1 g/kg (added to the fodder)
Sodium glutamate	160 mg per rat (as a 1.5% drink instead of water)
Glycine	12 mg per rat (added to the fodder)
*N*-Acetylcysteine	30 mg per rat (added to the fodder)
Vitamin C	4.4 mg per rat (added to the fodder)
Vitamin E	0.84 mg per rat (added to the fodder)
Selenium	4.0 mcg per rat (added to the fodder)
Commercial fish oil rich in vitamin A and omega 3 rich PUFA	1 drop per rat (sublingually)
Potassium iodide	4.0 mcg per rat (added to the fodder)

We gave glutamate to the rats as 1.5% solution instead of drinking water *ad libitum*. The “Amber Dew” (Ecco-Plus Ltd.: Zhukovskiy, Russia), a fish oil preparation rich in PUFA mainly of the ω-3 group (24%), was administered through gavage at a dose of 1 mL per rat. The apple pectin enterosorbent (Promavtomatika Ltd.: Belgorod, Russia) was added to the rats’ fodder in a quantity corresponding to a dose ca. 1000 mg/kg body weight. Commercial preparations of iodide, amino acids and vitamins available as tablets were crushed and added to another portion of the fodder in quantities corresponding to recommended daily intake of these micronutrients for rats (where such recommendations were known only for humans, a recalculation to the rat’s nutritional requirement was made based on the species’ standard metabolism ratio).

Taking into consideration that the standard balanced fodder presumably meets the normal nutritional requirements of a rat, we assumed that additional intake of the above-listed bioactive substances would meet the increased needs connected with the molecular mechanisms of metallic NPs toxicity. Nevertheless, it had to be checked whether or not such presumed overloading with them would evoke any unfavorable effects. That is why in our subchronic experiment one group of rats was administered the same BPC but was not exposed to any toxicant.

## 5. Conclusions

Our experiment has shown that a subchronic exposure of rats to spherical nanoparticles of nickel(II) and of manganese(II, III) oxide of similar size cause the development of moderately expressed subchronic intoxication, which in both cases is characterized by adverse changes in a great number of mostly the same functional indices and damage of essentially the same type to the microscopic structure of the internal organs. However, the majority of signs suggest that Mn_3_O_4_-NPs are more toxic than NiO-NPs in spite of the fact that the accumulation of manganese (both total and, especially, in the form of the NPs) in the organs rich in RES cells (liver and spleen) was substantially lower in response to the impact of Mn_3_O_4_-NPs than the accumulation of nickel in response to the impact of NiO-NPs. Quite probably, both the higher toxicity and the lower retention of the Mn_3_O_4_-NPs in the organism are connected with their higher solubility as compared with NiO-NPs.

At the same time, exposure to Mn_3_O_4_-NPs increases the total amount of manganese in the brain, and the effect of damage to the neurons of the caudate nucleus and the hippocampus caused by their action in a higher degree than by the action of NiO-NPs can be considered as toxicologically specific to manganese.

The type of combined toxicity of Mn_3_O_4_-NPs and NiO-NPs is different for different indices (additivity, synergism, and subadditivity), and its identification deserves special mathematical analysis. On the whole, however, it should be stated that such combined exposure caused a no less pronounced overall picture of intoxication, including damage to the brain structures, than exposure to Mn_3_O_4_-NPs alone did.

Substantial attenuation of almost all effects due to the combined exposure by the administration of a set of bioprotectors the actions of which are targeted at different hypothesized mechanisms of toxicity has promising prophylactic prospects but also presents theoretical interest, indirectly confirming both the causality of those effects and the mechanistic premises of their choice.
